# Supplementary material for: Is it inside my head? Characterization of sound externalization in schizophrenia
Source: PLoS One. 2026 Mar 16;21(3):e0345074. doi: 10.1371/journal.pone.0345074 (PMC12991231; doi:10.1371/journal.pone.0345074)
Supplement: S1 Table — (ZIP) [file pone.0345074.s001.zip › S1_Table.docx]

**Table S1. Estimated effects from the final binomial GLM predicting externalization ratings**

| **Predictor** | **OR** | **95% CI Lower** | **95% CI Upper** | **z** | **p** |
| --- | --- | --- | --- | --- | --- |
| **(Intercept)** | **0.108** | **0.082** | **0.139** | **-16.63** | **<0.001** |
| **patients - controls** | **2.134** | **1.651** | **2.761** | **5.78** | **<0.001** |
| **HRTF - diotic** | **37.021** | **27.662** | **49.931** | **23.98** | **<0.001** |
| **BRIR- diotic** | **50.738** | **37.469** | **69.268** | **25.06** | **<0.001** |
| **anger - neutral** | **2.744** | **1.993** | **3.797** | **6.14** | **<0.001** |
| disgust - neutral | 1.222 | 0.869 | 1.720 | 1.15 | 0.249 |
| **fear - neutral** | **2.357** | **1.708** | **3.269** | **5.18** | **<0.001** |
| happiness - neutral | 1.354 | 0.961 | 1.910 | 1.73 | 0.083 |
| sadness - neutral | 1.292 | 0.914 | 1.829 | 1.45 | 0.147 |
| **patients - controls * HRTF - diotic** | **0.157** | **0.126** | **0.195** | **-16.62** | **<0.001** |
| **patients - controls * BRIR - diotic** | **0.125** | **0.098** | **0.158** | **-17.18** | **<0.001** |
| **patients - controls * anger - neutral** | **0.649** | **0.473** | **0.888** | **-2.69** | **0.007** |
| patients - controls * disgust - neutral | 1.043 | 0.771 | 1.410 | 0.27 | 0.785 |
| patients - controls * fear - neutral | 0.810 | 0.592 | 1.106 | -1.32 | 0.186 |
| **patients - controls * happiness - neutral** | **0.649** | **0.475** | **0.886** | **-2.72** | **0.007** |
| patients - controls * sadness - neutral | 0.788 | 0.572 | 1.082 | -1.47 | 0.142 |
| HRTF - diotic * anger - neutral | 0.843 | 0.586 | 1.213 | -0.92 | 0.359 |
| BRIR - diotic * anger - neutral | 1.295 | 0.877 | 1.913 | 1.30 | 0.193 |
| HRTF - diotic * disgust - neutral | 0.951 | 0.658 | 1.374 | -0.27 | 0.790 |
| BRIR - diotic * disgust - neutral | 1.225 | 0.836 | 1.794 | 1.04 | 0.298 |
| HRTF - diotic * fear - neutral | 0.982 | 0.680 | 1.416 | -0.10 | 0.923 |
| BRIR - diotic * fear - neutral | 1.194 | 0.814 | 1.751 | 0.91 | 0.364 |
| HRTF - diotic * happiness - neutral | 1.305 | 0.895 | 1.902 | 1.38 | 0.166 |
| **BRIR - diotic * happiness - neutral** | **1.506** | **1.022** | **2.222** | **2.07** | **0.039** |
| **HRTF - diotic * sadness - neutral** | **1.472** | **1.007** | **2.151** | **2.00** | **0.046** |
| **BRIR - diotic * sadness - neutral** | **1.853** | **1.249** | **2.754** | **3.06** | **0.002** |

Note: Odds ratios (OR) with 95% confidence intervals (CI) are shown for each predictor and interaction retained in the final model. The z-statistic and associated p-value correspond to the test of each coefficient (β) against zero on the logit scale. The reference levels are as follows: *healthy* *controls* for the group factor, *diotic sounds* for the sound type factor, and *neutral* for the emotion factor. Statistical significance is set at p-values < .05.
