# Supplementary material for: Is it inside my head? Characterization of sound externalization in schizophrenia
Source: PLoS One. 2026 Mar 16;21(3):e0345074. doi: 10.1371/journal.pone.0345074 (PMC12991231; doi:10.1371/journal.pone.0345074)

S1 Fig. Externalization ratings (sound source perceived as outside (1) or inside (0) the head) for the three types of sound processing (Diotic; HRTF: Head Related Transfer Function; BRIR: Binaural Room Impulse Response) and the six emotional contents in healthy controls (n = 24, in blue-green) and patients with schizophrenia (n = 23, in pink). Results are displayed as mean in percent ± one standard deviation.

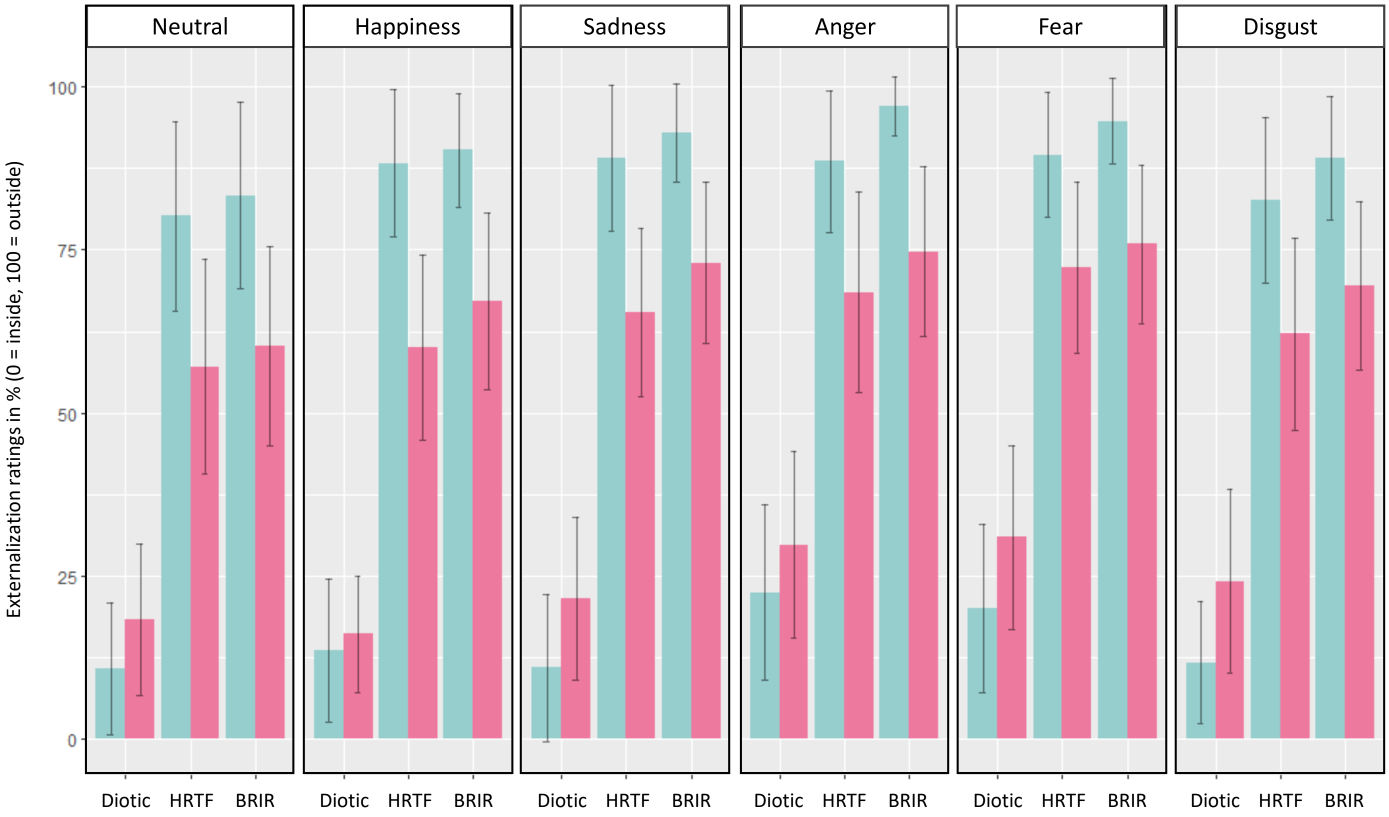

Supplement: S1 Table — (ZIP) [file pone.0345074.s001.zip › S1_Fig.pdf]
