# Supplementary material for: Is it inside my head? Characterization of sound externalization in schizophrenia
Source: PLoS One. 2026 Mar 16;21(3):e0345074. doi: 10.1371/journal.pone.0345074 (PMC12991231; doi:10.1371/journal.pone.0345074)

S2 Fig. Scatterplots of the patients' symptomatology scores as a function of the externalization ratings across the three types of processing (Diotic; HRTF: *Head Related Transfer Function*; BRIR: *Binaural Room Impulse Response*). Scatterplots are plotted for each of the correlations leading to a Spearman coefficient with an absolute value above 0.4. Note that none of these correlations was significant after Bonferroni correction (α=0.002). PANSS: *Positive and negative syndrome scale*; SAPS: *Scale for the Assessment of Positive Symptoms*.

Figure 2A. Scatterplot of the negative symptoms as a function of the externalization ratings

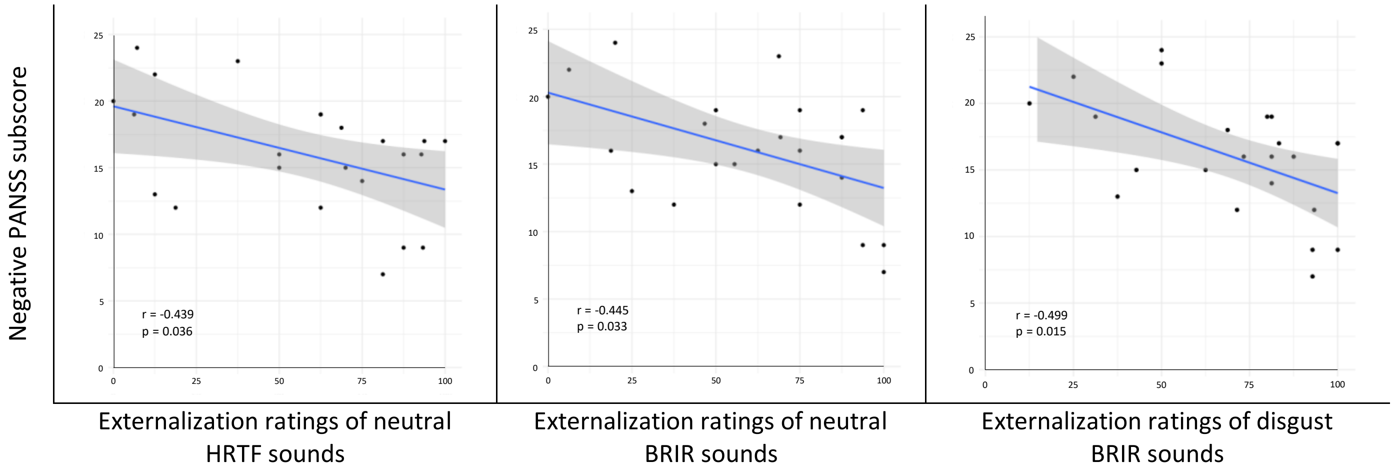

Figure 2B. Scatterplot of the positive symptoms as a function of the externalization ratings

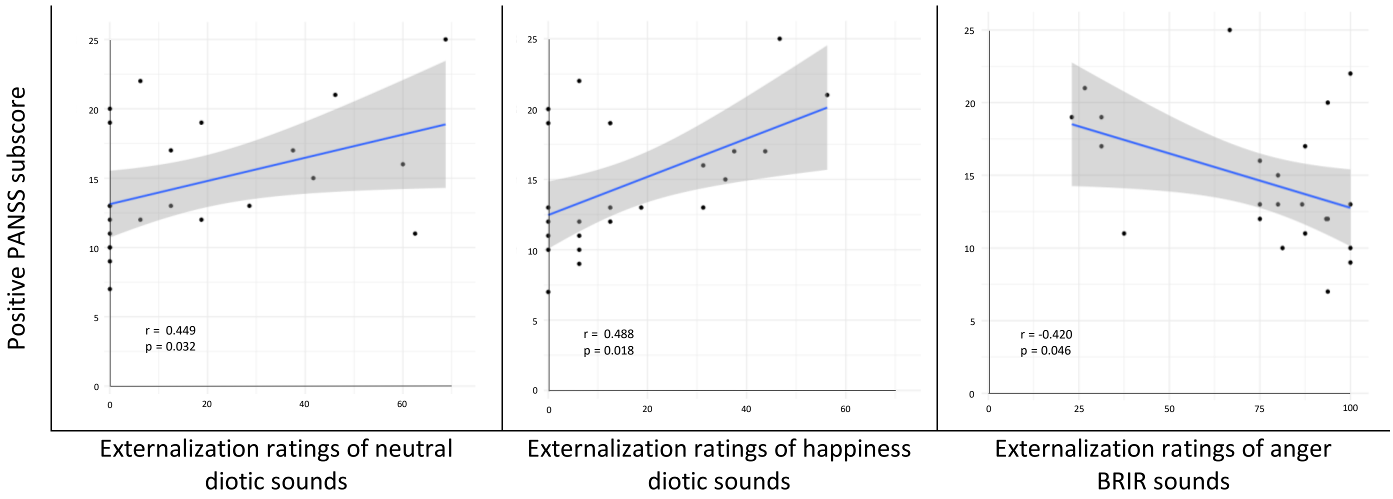

Figure 2C. Scatterplot of the hallucinations as a function of the externalization ratings

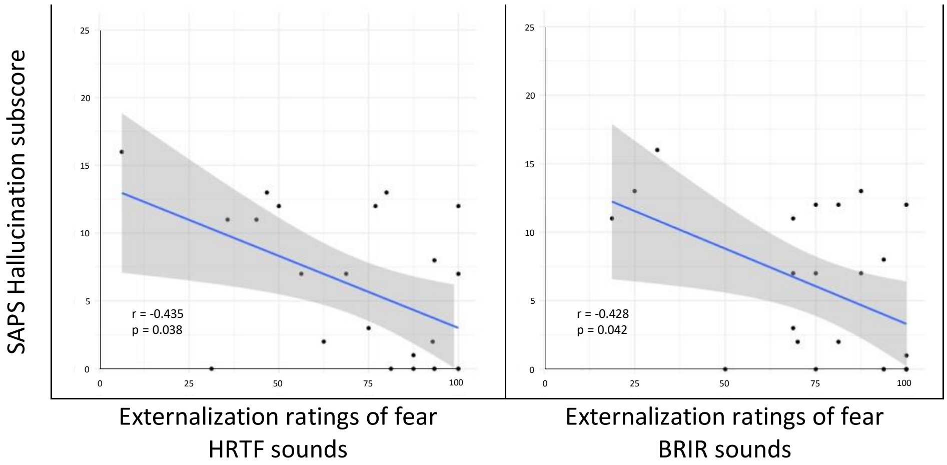

Supplement: S1 Table — (ZIP) [file pone.0345074.s001.zip › S2_Fig.pdf]
